# Defining the Human Brain Proteome Using Transcriptomics and Antibody-Based Profiling with a Focus on the Cerebral Cortex

**DOI:** 10.1371/journal.pone.0130028

**Published:** 2015-06-15

**Authors:** Evelina Sjöstedt, Linn Fagerberg, Björn M. Hallström, Anna Häggmark, Nicholas Mitsios, Peter Nilsson, Fredrik Pontén, Tomas Hökfelt, Mathias Uhlén, Jan Mulder

**Affiliations:** 1 Science for Life Laboratory, School of Biotechnology, KTH-Royal Institute of Technology, Stockholm, Sweden; 2 Science for Life Laboratory, Department of Immunology, Genetics and Pathology, Uppsala University, Uppsala, Sweden; 3 Science for Life Laboratory, Department of Neuroscience, Karolinska Institute, Stockholm, Sweden; Medical College of Wisconsin, UNITED STATES

## Abstract

The mammalian brain is a complex organ composed of many specialized cells, harboring sets of both common, widely distributed, as well as specialized and discretely localized proteins. Here we focus on the human brain, utilizing transcriptomics and public available Human Protein Atlas (HPA) data to analyze brain-enriched (frontal cortex) polyadenylated messenger RNA and long non-coding RNA and generate a genome-wide draft of global and cellular expression patterns of the brain. Based on transcriptomics analysis of altogether 27 tissues, we have estimated that approximately 3% (n=571) of all protein coding genes and 13% (n=87) of the long non-coding genes expressed in the human brain are enriched, having at least five times higher expression levels in brain as compared to any of the other analyzed peripheral tissues. Based on gene ontology analysis and detailed annotation using antibody-based tissue micro array analysis of the corresponding proteins, we found the majority of brain-enriched protein coding genes to be expressed in astrocytes, oligodendrocytes or in neurons with molecular properties linked to synaptic transmission and brain development. Detailed analysis of the transcripts and the genetic landscape of brain-enriched coding and non-coding genes revealed brain-enriched splice variants. Several clusters of neighboring brain-enriched genes were also identified, suggesting regulation of gene expression on the chromatin level. This multi-angle approach uncovered the brain-enriched transcriptome and linked genes to cell types and functions, providing novel insights into the molecular foundation of this highly specialized organ.

## Introduction

The brain is a complex organ that controls a variety of bodily functions, including maintenance of homeostasis, processing of sensory information, cognition and generation of behaviors. These functions are executed by circuitries composed of specialized neurons supported by glial cells (astrocytes, oligodendrocytes and microglia) that each express sets of genes that determine their phenotype and physiological properties.

The human genome projects [1,2] revealed the genetic code, enabling extensive analysis of gene expression in tissue and organ samples in the context of evolution, physiology and disease. The majority of these data, including >35,000 data sets linked to human brain, are published in online repositories such as the Gene Expression Omnibus [3] and ArrayExpress [4]. This huge amount of expression data and the development of next generation sequencing technologies have opened venues to explore gene expression, regulation of gene expression, splice variation and gene function on organ, tissue and cellular level. In fact, a meta analysis of public available data of >200 different studies using the Affymetrix U133A microarray platform generated the first global map of human gene expression [5]. The introduction of RNAseq platforms has enabled more thorough and faster genome wide expression analyses of various tissues available in the ensembl database [6] and Genotype-Tissue Expression (GTEx) portal [7] as well as peer-reviewed, whole genome deep sequencing studies comparing 11 [8] and 27 [9,10] tissue and organ types, including brain. These resources provide a detailed documentation of global gene expression and have identified ubiquitous versus more organ specific genes, showing the highest numbers of tissue-enriched genes to be expressed in testis and brain.

It has recently become evident that subsets of long non-coding RNAs (lncRNAs) regulate transcription and translation as precursor of microRNAs, by binding to microRNAs or interacting with microRNA binding sites [11], by chromatin modifications [12] and by interacting with genetic elements that enhance gene expression [13]. Like mRNA, lncRNA are RNA polymerase II products, containing a 5’ cap and poly A tail and are frequently spliced [14]. Ensembl version 73 annotates and reports 6,969 lncRNA-coding genes, and the GENECODE consortium annotated 9,277 lncRNA coding genes producing 14,800 transcripts [15]. The brain expresses the highest levels of non-coding RNA when comparing 12 tissues (testis not included) [16], and Kim and colleagues [13] found a correlation between levels of enhancer RNA and levels of mRNA synthesized by neighboring genes in mouse cortical neurons. These data suggest an organ-specific regional organization of chromatin structures or presence of other epigenetic mechanisms that regulate transcription of clustered coding and non-coding genes.

Here we analyzed genes expressed in a functionally important area of the human brain, the frontal cortex (FC). By comparing 27 tissue types representing all major organs and tissues in the human body, brain-enriched protein coding [9] and non-coding genes could be filtered, enabling a detailed survey of expression patterns and specialized biological processes specific for brain. Transcriptomics, gene ontology analysis and detailed evaluation of immunohistochemistry (IHC) results were combined to create a unique view on brain-enriched genes important for cortical physiology and provide insights in the genetic molecular mechanisms of gene expression in the brain.

## Results

### The Human transcriptome

**Fig 1:**
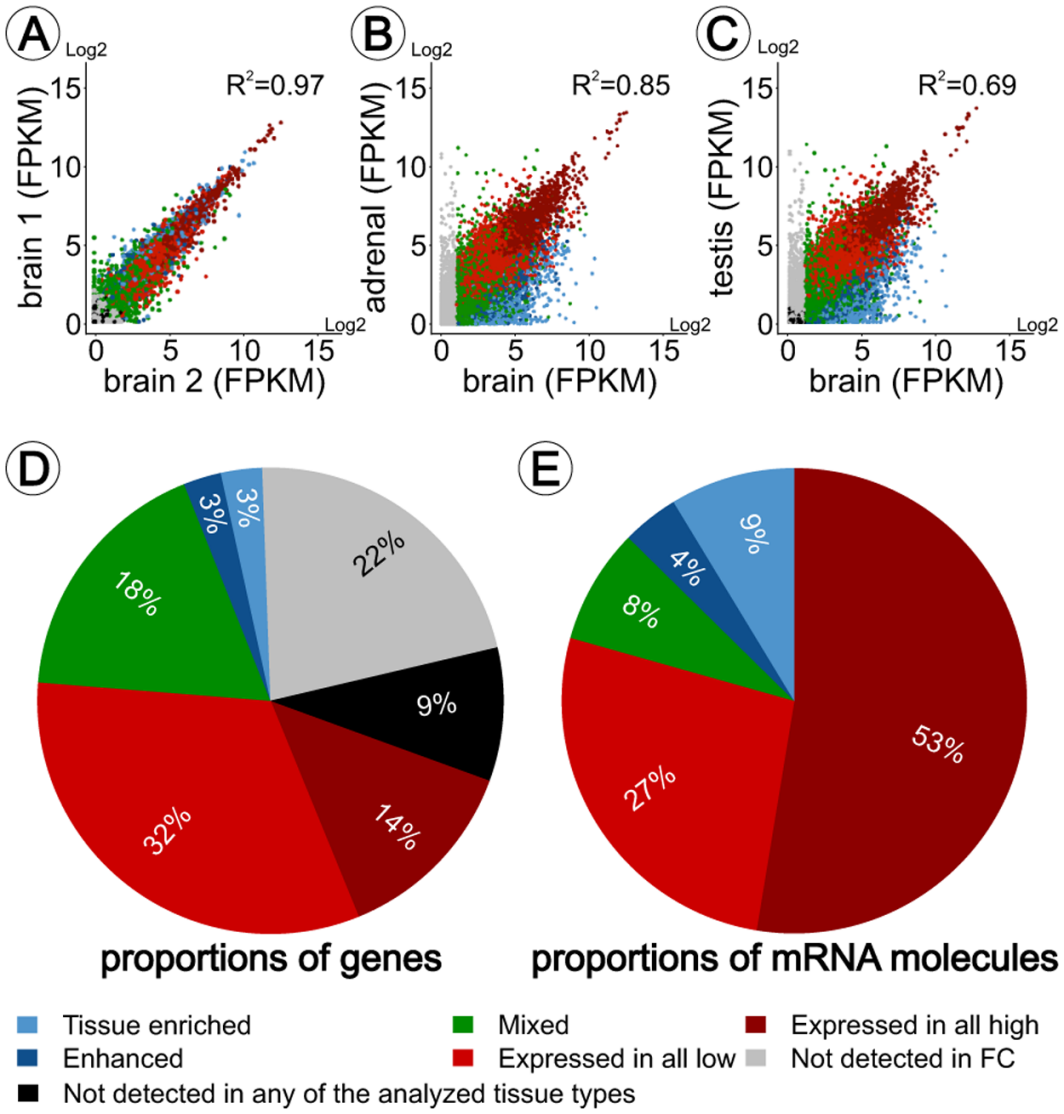
Transcriptome sequencing of brain tissue. Scatter plot with Spearman correlation analysis between individual brain samples (A), between brain and the most similar peripheral tissue, the adrenal gland (B) and between brain and the least similar tissue, testis (C). 69% of all 20,239 currently known genes are expressed in the frontal cortex (FPKM >1). The majority of these genes are highly (14%) or moderately (32%) expressed in all tissue types (D). The majority (80%) of mRNA molecules in frontal cortex codes for proteins expressed in most tissue types analyzed, 4% of detected transcripts are brain-enhanced and 9% are brain-enriched (E).

The transcriptomes of 26 peripheral human organs (testis, bone marrow, kidney, liver, esophagus, skin, heart, adrenal gland, adipose tissue, endometrium, ovary, pancreas, thyroid gland, prostate, salivary gland, stomach, colon, small intestine, duodenum, placenta, spleen, lymph node, appendix, lung, gall bladder, urinary bladder) and three frontal cortex samples (S1 Fig) were analyzed using next generation sequencing based on specimens from altogether 95 individuals [9]. The transcriptome of each sample was quantified using RNAseq to determine the normalized mRNA abundance, calculated as fragments per kilobase of exon model per million mapped reads (FPKM) [17]. In these analyses we used a cut-off value of 1 FPKM, that roughly represents one mRNA molecule per average cell in the sample [18]. High correlation between biological replicates (Fig 1A) indicates low inter-individual variability in gene expression between the three frontal cortex samples. Correlation analysis also revealed relationships between tissue types. For example, both brain and adrenal medulla originate from the neuro-ectoderm, and in agreement the correlation coefficients between brain and adrenal gland is higher (Fig 1B) as compared to brain versus testis (Fig 1C). Validation was carried out by comparing transcriptomics data generated in this project with public available data from the BrainSpan project (http://www.brainspan.org). This analysis revealed a correlation coefficient >0.9, indicating reproducibility of sequencing experiments in general.

### Classification of protein coding genes in the brain

Genes expressed in the frontal cortex were classified according the categories determined to describe expression profiles in a large set of related and unrelated tissues [9,19–24]. In total 13,992 of all known genes are expressed (FPKM >1) in the frontal cortex (Fig 1D). The majority of these genes is highly or moderately expressed in all tissue types analyzed (Fig 1E). In total 3,594 genes are expressed in the majority, but not all analyzed tissue types, and represent a mix of low abundant housekeeping genes and specialized genes expressed in many tissues. Comparing brain to global expression we identified 1,113 enhanced-genes (6%; S1 Table) with at least a 5-fold higher expression in brain compared to the average expression in all peripheral tissues. This analysis also revealed 21 highly enriched (>50-fold higher FPKM level in brain compared to any other tissue), 302 genes moderately enriched (>5-fold higher FPKM level in brain compared to any other tissue) and 248 group-enriched genes (> 5-fold higher average FPKM level in a group of 2–7 tissues including brain compared to other tissues). The brain-enriched transcriptome consists of approximately 3% (571 genes) of all genes (FDR <5%; S1 Table). Approximately 13% of the total mRNA is encoded by brain-enhanced and brain-enriched genes (Fig 1E).

### Gene Ontology analysis

**Fig 2:**
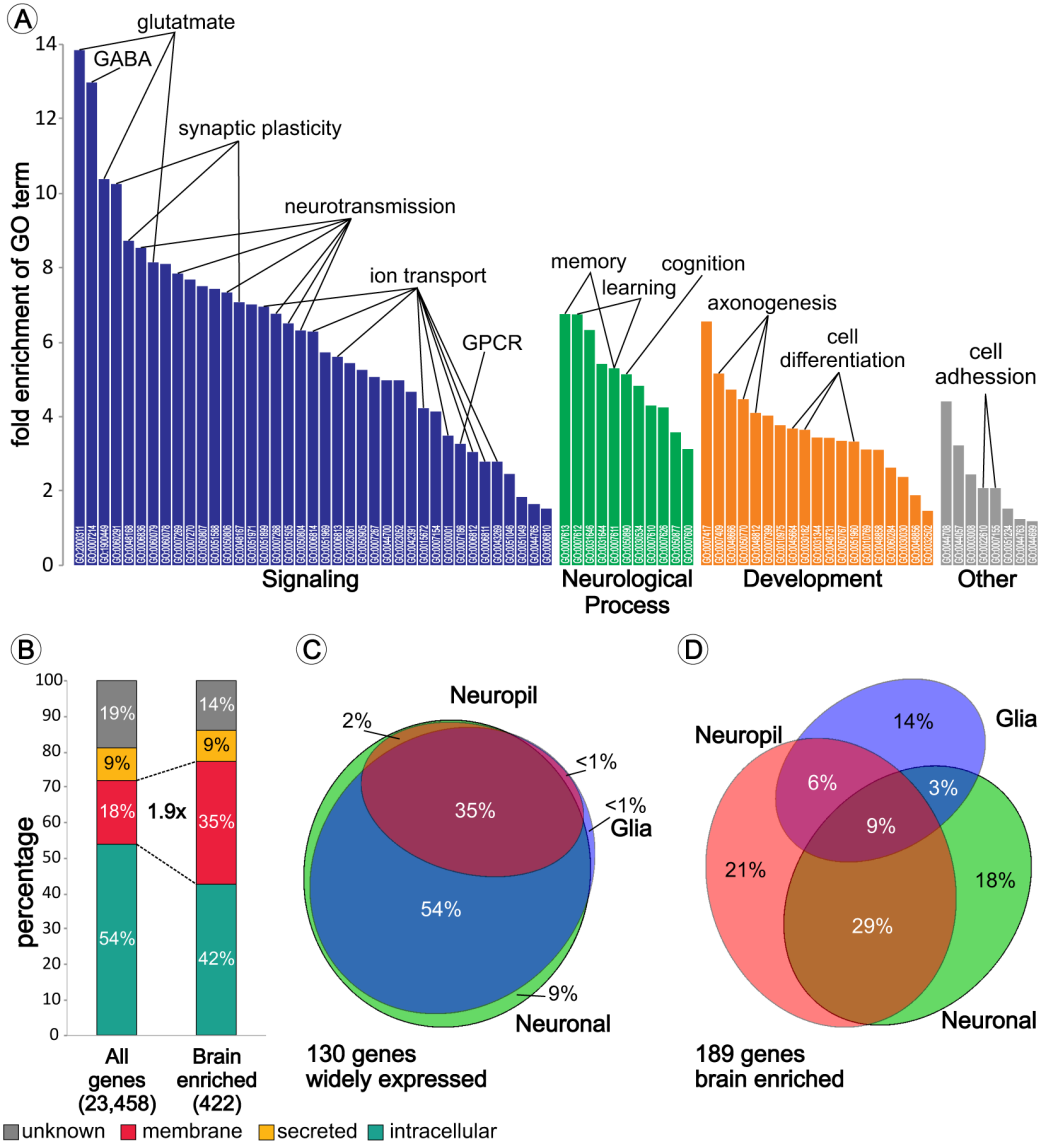
Brain-enriched genes function, cellular and subcellular location. Graphical representation of enriched GO-terms sorted according to process and fold enrichment (A). GO terms and statistics are listed in S3 Table. GOslim analysis of all genes versus 422 brain-enriched genes revealed a 1.9x enrichment of membrane bound proteins (B). VENN diagram showing the cellular distribution of 130 randomly selected genes expressed in all tissues (C) and 190 brain-enriched genes in neurons, glial cells and neuropil (containing pre- and post-synaptic compartments and glial endfeet) (D).

To categorize the specialized cellular and physiological functions of brain-enriched protein-coding genes, we performed a GO analysis using all genes expressed in the frontal cortex as background expression. Seventy-seven processes with a significant enrichment (p<10^–6^) were found (Fig 2A and S3 Table). The majority of enriched processes are linked to various aspects of synaptic signaling or neurological processes driven by cortical neuronal circuits. In addition we identified a cluster of brain-enriched developmental processes, many of which are specific for development of the nervous system. Furthermore, cellular component analysis, using GO-Slim tools, revealed a 1.9x enrichment of proteins located in the cell membrane (Fig 2B). This indicates that specialized brain proteins are more commonly membrane and extracellular proteins involved in synaptic functions and developmental processes.

### Antibody-based analysis of the brain-enriched genes

Immunohistochemistry (IHC) results for >300 proteins, including 190 brain-enriched proteins (S4 Table), were analyzed to determine the cellular (neurons, glial cells, endothelial cells) and subcellular (neuropil, soma, nucleus) distribution of corresponding proteins. The majority of widely expressed proteins are located in both glia and neurons, located in multiple cellular compartments (Fig 2C). Brain-enriched proteins are more often selectively expressed in either neurons or glial cells and are in many instances only found in the neuropil (Fig 2D). These findings are supported by expression profiles of sorted neurons, glia cells and vascular cells from the mouse cerebral cortex [25], showing clustering of human brain-enriched genes in specific cell types (S2 Fig).

**Fig 3:**
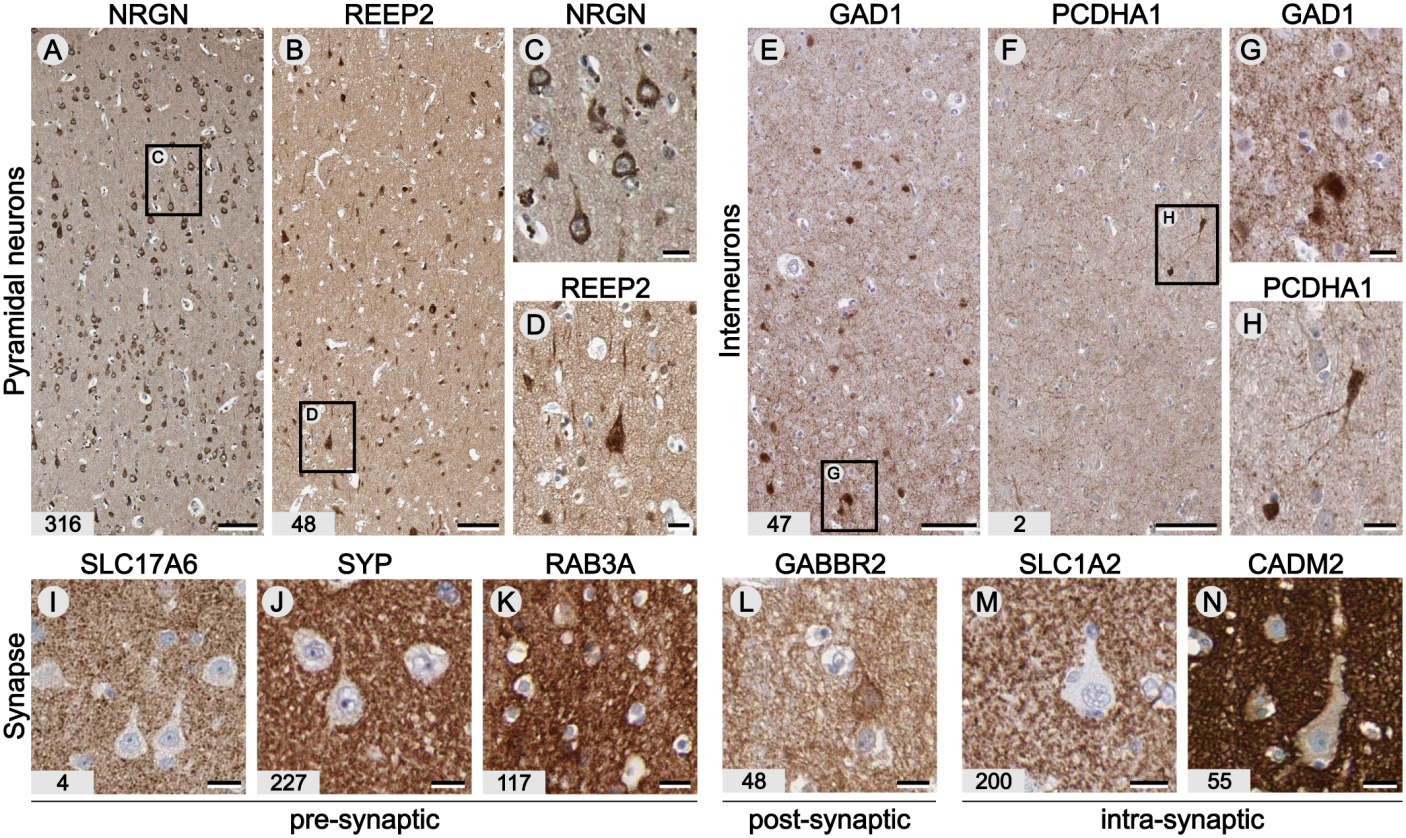
Proteins expressed in neurons and localized at the synapse. An antibodies against neurogranin (NRGN) and receptor expression-enhancing protein 2 (REEP2) label pyramidal-like neurons (A,C). Note the difference in the number of neurons immunoreactive for NRGN and REEP2 (B,D). Most inhibitory GABAergic neurons in the cerebral cortex express glutamate decarboxylase 1 (GAD1 or GAD67; E,G). An antibody against protocadherin alpha-1 shows immunoreactivity in a subset of mainly bipolar interneuron-like cells (F,H). We also included IHC results of brain-enriched genes known to be located in the presynaptic terminal including vesicular glutamate transporter 2 (SLC17A6; I), synaptophysin (SYP; J) and Ras-related protein RAB3A (K) the postsynaptic protein GABA type B receptor subunit 2 (GABBR2; L) and the intra-synaptic proteins excitatory amino acid transporter 2 (SLC1A2) expressed by astrocytes (M) and cell adhesion molecule 2 (CADM2; N). Corresponding FPKM values are displayed in the bottom left corner. Scale bars: A,B,E,F = 100 μm; C,D & G-N = 20 μm.

In cortical regions the neuronal population is roughly divided into two main classes, excitatory, glutamatergic pyramidal projection neurons (≈75%) and inhibitory, mostly GABAergic interneurons (≈25%). Among the list of brain-enriched genes we found several proteins expressed in pyramidal-like neurons including widely expressed neurogranin (NRGN; Fig 3A and 3C) [26] and the less well-characterized receptor expression enhancing protein 2 (REEP2) (Fig 3B and 3D) and genes expressed in cortical interneurons like glutamate decarboxylase 1 (GAD1 or GAD67) [27] (Fig 3E and 3G) and protocadherin alpha-1 (PCDHA1) located in a subpopulation of interneuron-like cells (Fig 3F and 3H). However, the majority of brain-enriched genes could not be found in neuronal cell bodies. Neuronal proteins involved in synaptic neurotransmission are, after ribosomal synthesis, rapidly centrifugally transported to the nerve endings or translated locally in the postsynaptic terminal. The brain-enriched genes include several proteins known to be involved presynaptic functions (Fig 3I–3K), including vesicular glutamate transporter 2 (SLC17A6 or VGLUT2) [28], synaptophysin (SYP) and Ras-related protein RAB3A involved in exocytosis [29], post-synaptic proteins including the GABA B receptor subunit 2 (GABBR2, Fig 3L) [30] and intrasynaptic excitatory amino acid transporter 2 (EAAT2 or SLC1A2, Fig 3M) [31] and cell adhesion molecule 2 (CADM2 or SYNCAM2, Fig 3N) [32]. These findings indicate that all aspects of synaptic transmission involve specialized proteins not expressed in tissues lacking exocytosis driven cellular communication.

**Fig 4:**
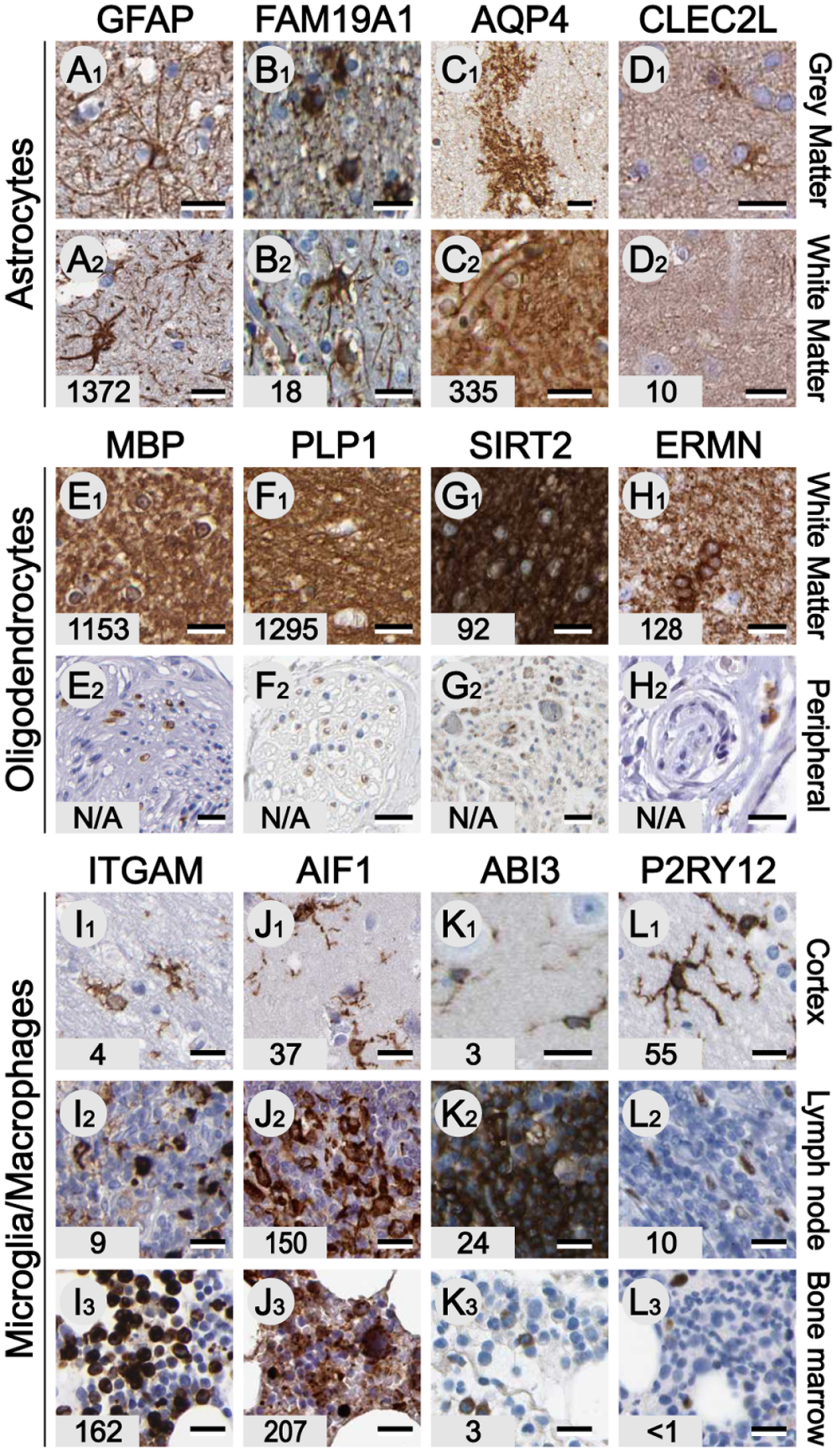
Proteins expressed in glial cells. Glial fibrillary acidic protein (GFAP), FAM19A1, Aquaporin-4 (AQP4) and C-type lectin domain family 2 member L (CLEC2L) are expressed by astrocyte like cells (A-D). Note the difference in expression between astrocytes populating white and grey matter. Compact myelin components myelin basic protein (MBP) and proteolipid protein (PLP1) and components of non-compact myelin including NAD-dependent protein deacetylase sirtuin-2 (SIRT2) and ermin (ERMN) are highly expressed in white matter (E1-H1) and peripheral nerves (E2-H2). The majority of microglia genes, including Integrin alpha-M (ITGAM), a.k.a. CD11B (I), allograft inflammatory factor 1 (AIF1) a.k.a. IBA1 (J) and ABI gene family member 3 (ABI3; K), are highly expressed by various monocytes and monoblasts in lymph node (I2-K2) as well as bone marrow (I3-K3). P2Y, purinoceptor 12, is the only brain-enriched microglia gene found. Corresponding FPKM values are displayed in the bottom left corner. Scale bars: A-L = 20 μm.

Glial cells constitute the most numerous class of cells in the brain and can generally be subdivided into astrocytes, oligodendrocytes and microglia based on morphology and function. In line with that, the top three most abundantly expressed (FPKM > 1,000) brain-enriched genes in the analyzed samples (S1 Table) are genes expressed by glial cells. The majority of glial cells immunoreactive for antibodies raised against brain-enriched genes has an astrocyte-like staining pattern and are found in both grey and white matter. However, variation in distribution, morphology and cell density was observed. The general astrocyte marker GFAP (Fig 4A) [33] and the unexplored gene FAM19A1 (Fig 4B) with sequence similarity to chemokines are expressed in astrocyte-like cells in both the white and grey matter. In contrast aquaporin 4(AQP4; Fig 4C) is mainly located in the grey matter, and antibodies against AQP4 reveal a neuropil-like staining pattern due to the localization of this protein in numerous glial end feet [34]. The uncharacterized lectin-like protein (C-type lectin domain family 2, member L, CLEC2L; Fig 4D) is mainly localized in astrocyte-like cells in the white matter. These data illustrate the heterogeneity among astrocytes and the need to further classify astrocytes based on morphology, location and gene expression in order to understand the various specialized functions of astrocytes.

We also investigated the global expression of molecular components of myelin expressed in oligodendrocytes [35], including the compact myelin proteins myelin basic protein (MBP, Fig 4E) and proteolipid protein 1 (PLP1; Fig 4F) as well as components of the non-compact myelin such as sirtuin 2 (SIRT2; Fig 4G) and ermin (ERMN; Fig 4H). MBP and PLP1 are highly brain-enriched, mainly due to the sample composition containing 25% densely myelinated white matter, but also FPKM values >10 were found in many tissue types. Examination of IHC revealed that this expression mainly represents Schwann cells in peripheral nerves (Fig 4E2–4G2).

The third class of glial cells ‘populating’ the brain is microglia. These cells derived from hematopoietic stem cells that invaded the brain during embryonic development or macrophages that entered the brain from the bloodstream later in life. All well known microglia genes, including integrin alpha M chain (ITGAM or CD11b; Fig 4I) [36] and allograft inflammatory factor 1 (AIF1 or IBA1; Fig 4J) [37], are also highly expressed in cells in the lymph nodes (Fig 4I2–4J2) and bone marrow (Fig 4I3–4J3), the main sites of hematopoiesis. The less well-characterized ABI gene family member 3 (ABI3 or NESH) is expressed in brain microglia and lymph nodes, with relatively low abundance in bone marrow (Fig 4K). Based on our IHC analysis we could only identify one microglia gene, purinoceptor P2RY12, enriched in brain tissue and with lower expression in lymph nodes and bone marrow (Fig 4L).

**Fig 5:**
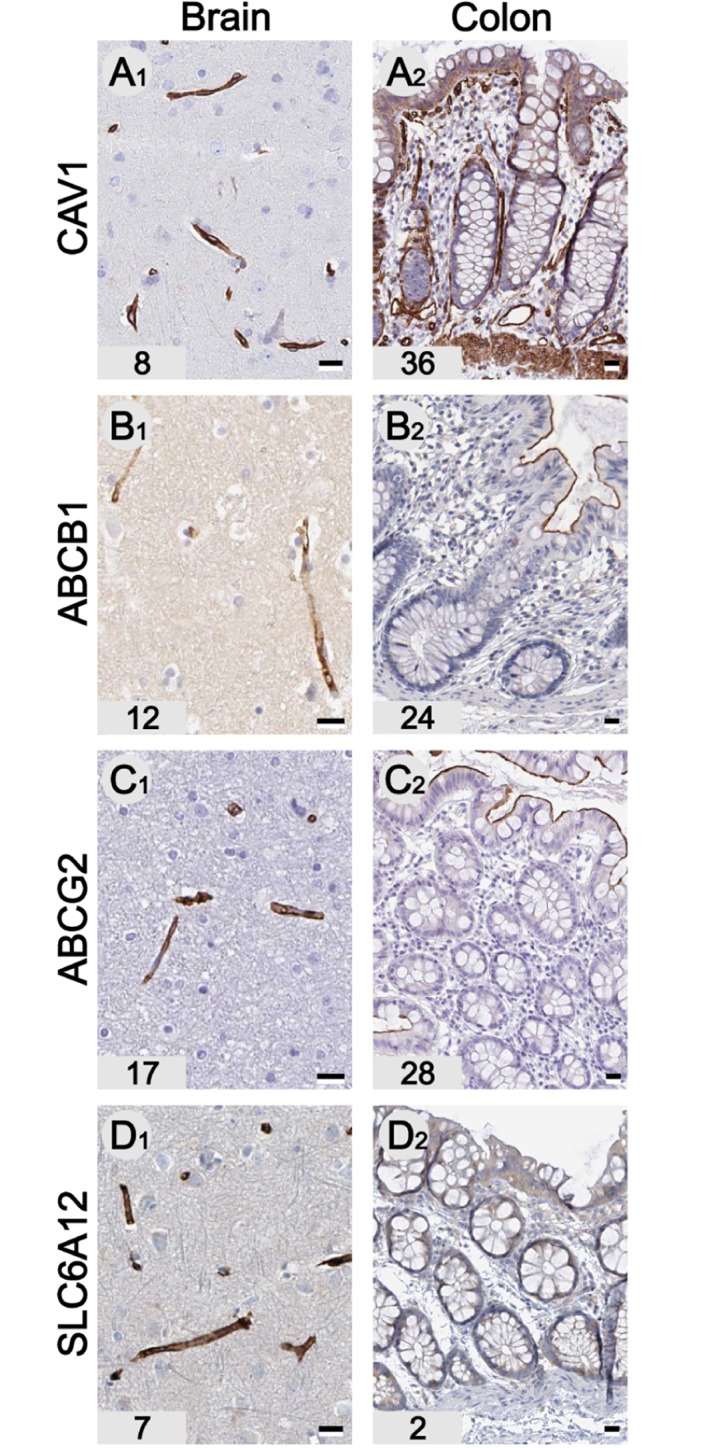
Proteins expressed in endothelial cells. Caveolin-1 is a scaffolding protein within caveolar membranes and is expressed in endothelial cells in all tissue types including brain (A1) and colon (A2) The BBB proteins P-glycoprotein 1 (ABCB1) and Breast cancer resistance protein (ABCG2) are expressed by brain-endothelial cells (B1, C1). Antibodies against both ABC transporters show no immunoreactivity in capillaries in colon, but both show immunoreactivity in the columnar epithelium (B2,C2). SLC6A12 is a sodium- and chloride-dependent betaine transporter, which is brain-enriched and shows immunoreactivity only in brain capillaries (D). Corresponding FPKM values are displayed in the bottom left corner. Scale bars: A-D = 20 μm.

In addition to neurons and glial cells, a dense network of blood vessels and capillaries (5% of total brain volume) provides the brain with sufficient oxygen and glucose. Several genes generally expressed by endothelial cells such as the membrane protein caveolin 1 (CAV1) are expressed in blood vessels [38] in all organs including brain and colon (Fig 5A). To shield the brain from influx of neurotoxic molecules of various types, the capillaries in the central nervous system express several efflux pumps that form the blood-brain-barrier [39]. The two main components of this brain specific feature are the ATP-binding cassette sub-family transporters P-glycoprotein 1 (P-gp, ABCB1) and breast cancer resistance protein (BCRP, ABCG2). Unlike their organ function these efflux pumps are not brain-enriched, but expressed in many tissues including colon (Fig 5B and 5C). In colon tissue these ABC transporters are not expressed in endothelial cells, but in the glandular cells of the columnar epithelium [40], suggesting a different transport function in brain and colon. Based on IHC and RNAseq data, only one brain-enriched gene expressed by endothelial cells was identified: the solute carrier family protein SLC6A12 (GAT-2 or BGT-1), which is a betaine and GABA transporter that is brain-enriched and expressed in brain capillaries (Fig 5D) [41]. It implies a possible role of SLC6A12 in drug resistance epilepsy as many of the drugs for the treatment of seizures target GABA transporters. These data illustrate and confirm that the majority of brain-enriched genes are cell-type specific most commonly expressed in neurons, astrocytes and oligodendrocytes. Microglia and endothelial cells share the majority of expressed genes with related cells in other organs. However, both endothelial cells and microglia express brain-enriched genes, suggesting adaptation of these cells to perform their specialized functions in the central nervous system.

### Group-enriched genes

**Fig 6:**
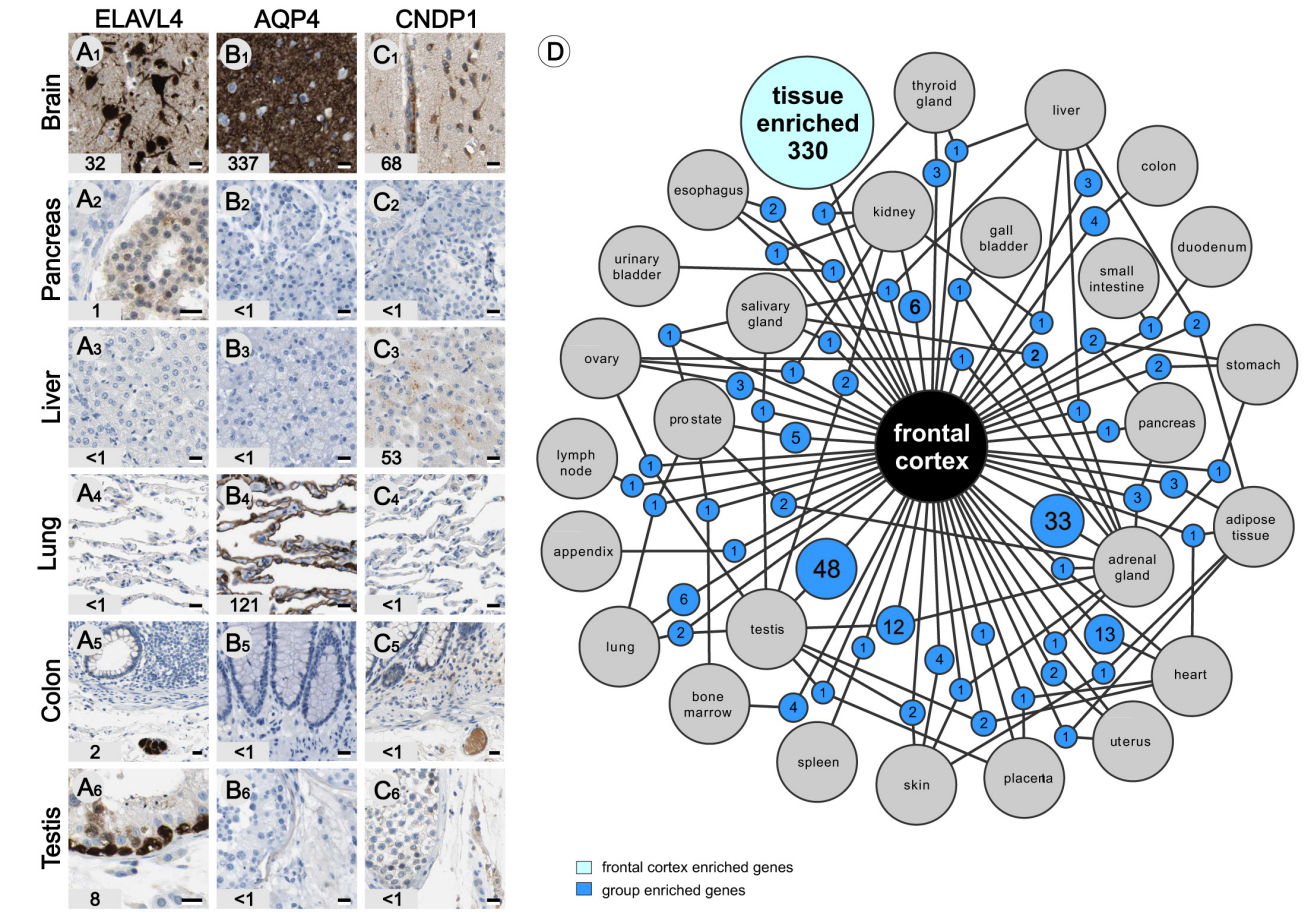
Group-enriched genes. Group-enriched genes are non-housekeeping genes with enriched (>5x) expression in 2–7 organ and tissue types. ELAV-like protein 4 (A) involved in neuron specific RNA processing is expressed in the central (A1) and peripheral neurons (A5) and pancreatic beta cells also originating from the neural crest (A2). In addition ELAVL4 immunoreactivity was found the testis (A6). IHC analysis reveals expression of AQP4 in astrocytes (B1) in the brain and pneumocytes cells in the lung (C4). Beta-Ala-His dipeptidase (CNDP1) is expressed in brain and liver. Antibodies raised against CNDP1 label neurons (C1) and plasma in the lumen of blood vessels (C1, C5, C6) in many tissues. Note the weak immunoreactivity of our CNDP1 antibody in liver (C3), probably indicating fast secretion of this dipeptidase into the bloodstream. A network plot (D) showing the distribution of group-enriched genes expressed in brain and 26 other organ and tissue types (due to complexity only 4 out maximal 7 levels are presented). Corresponding FPKM values are displayed in the bottom left corner. Scale bars: A-C = 20 μm.

During the initial analysis of the deep sequence data several genes enriched in a limited number (2–7) of tissue types were found. These are clearly non-housekeeping proteins that can have either similar or more diverse functions in different tissues. Several group-enriched genes are expressed in peripheral organs that contain cell types that originate from the neural crest, a developmental structure closely related to the neuro-ectoderm that forms the brain. For example, ELAV (embryonic lethal, abnormal vision)-like 4 is expressed in peripheral ganglion cells (Fig 6A5) and endocrine pancreas (Fig 6A2), both of which are derived from the neural crest. However, like many previously established ‘brain’ proteins, this molecule is also expressed in spermatogonia in the testis (Fig 6A6), having very little in common with neurons, suggesting multiple functions of this protein. Aquaporin 4 (AQP4) is a well-characterized water transporter involved in maintaining water homeostasis and is highly expressed in brain (Fig 6B1) and lungs (Fig 6B4). A more complex example of a group-enriched gene is Beta-Ala-His dipeptidase (CNDP1), a gene expressed in the brain (Fig 6C1) and liver (Fig 6C3). In the brain this small dipeptide-hydrolyzing enzyme is located in neurons and the lumen of capillaries and blood vessels (Fig 6C1). However, in the liver only traces of this secreted protein can be detected in hepatocytes using IHC (Fig 6C3), whereas strong labeling of blood vessel lumen (plasma) is seen in all analyzed organs and tissues (Fig 6C5–6C6). These examples illustrate the complexity that underlies biological functions, as some enriched genes are expressed in cells with shared origin and physiology, whereas other group-enriched proteins are expressed in unrelated cells with specific organ functions.

### Splice variation and non-coding transcripts

**Fig 7:**
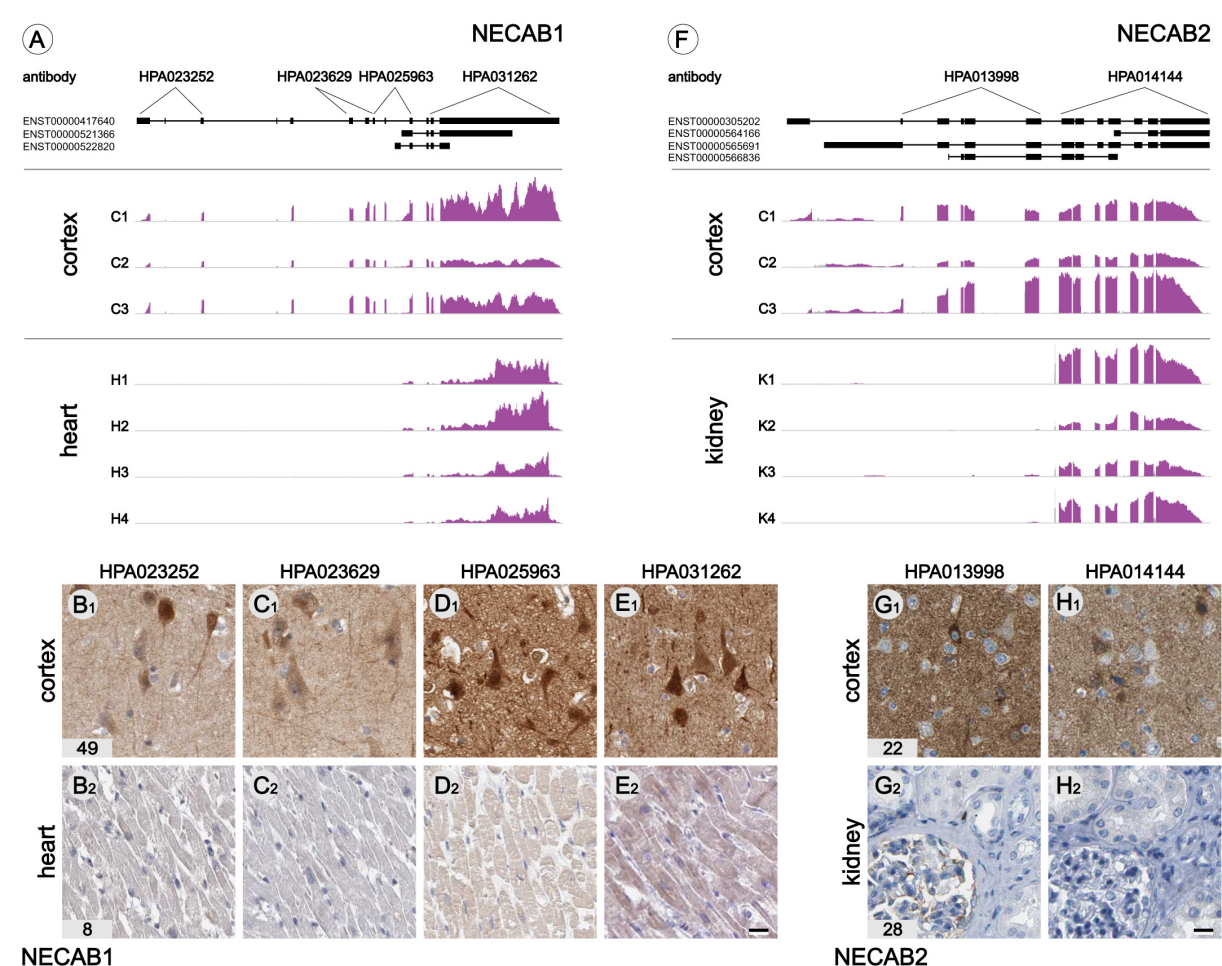
Tissue-enriched splice variants. Examples of alternative splice variants expressed in different tissues. Detailed analysis of mapped reads revealed the absence of exon 1–8 in heart muscle and the expression of transcript ENST00000521366 (A). The presence of the full and truncated protein-coding transcript in brain and heart was determined using 4 antibodies raised against different portions of NECAB1. Antibodies that only recognize full size NECAB1 show immunoreactivity in brain only (B-C). HPA031262 recognizing the C-terminal portion of NECAB1 binds to both the full size and truncated form of NECAB1 and gives a moderate immunoreactivity in heart muscle (E). NECAB2 has 4 known protein-coding transcripts of which the two N-terminal transcripts (ENST00000305202 and ENST00000565691) are expressed in brain and not in kidney (F). RNAseq data suggest the expression of a splice variant in kidney that does not match any of the protein coding transcripts supported by the lack of NECAB1 immunoreactivity in kidney (G,H). Corresponding FPKM values are displayed in the bottom left corner. Scale bars: B-E = G,H = 20 μm. Graphical representations of reads mapped to all known transcripts (89,933) for all analyzed tissue and organ samples are available at the protein atlas portal (http://www.proteinatlas.org

RNA-sequencing reveals the full mappable sequence of available transcripts and can be utilized to identify splice variants coded from each gene. For example, different transcripts for N-terminal EF-hand calcium binding proteins (NECABs) 1 and 2 are expressed in different organs. NECAB1 is brain-enriched (FPKM 49) but also expressed in heart muscle (FPKM 8). Based on the sequence data it is evident that the full-length transcript (ENST00000417640) is expressed in the brain, whereas a truncated transcript (ENST00000521366) is predominantly expressed in heart muscle (Fig 7A). This was supported by IHC analysis, where only the antibody targeting epitopes present in both isoforms stained both brain and weakly labeled heart muscle (Fig 7B–7E). A similar pattern was observed for NECAB2 when comparing brain and kidney; also, for NECAB2 the full-length transcript (ENST00000305202) was found in brain (Fig 7F). In kidney our transcriptome data suggest the existence of a truncated form of NECAB2, however the reads do not map to any of the known protein-coding transcripts, and no evidence for the existence of NECAB2 protein in kidney was found (Fig 7G and 7H).

### Brain-enriched long non-coding RNA

**Fig 8:**
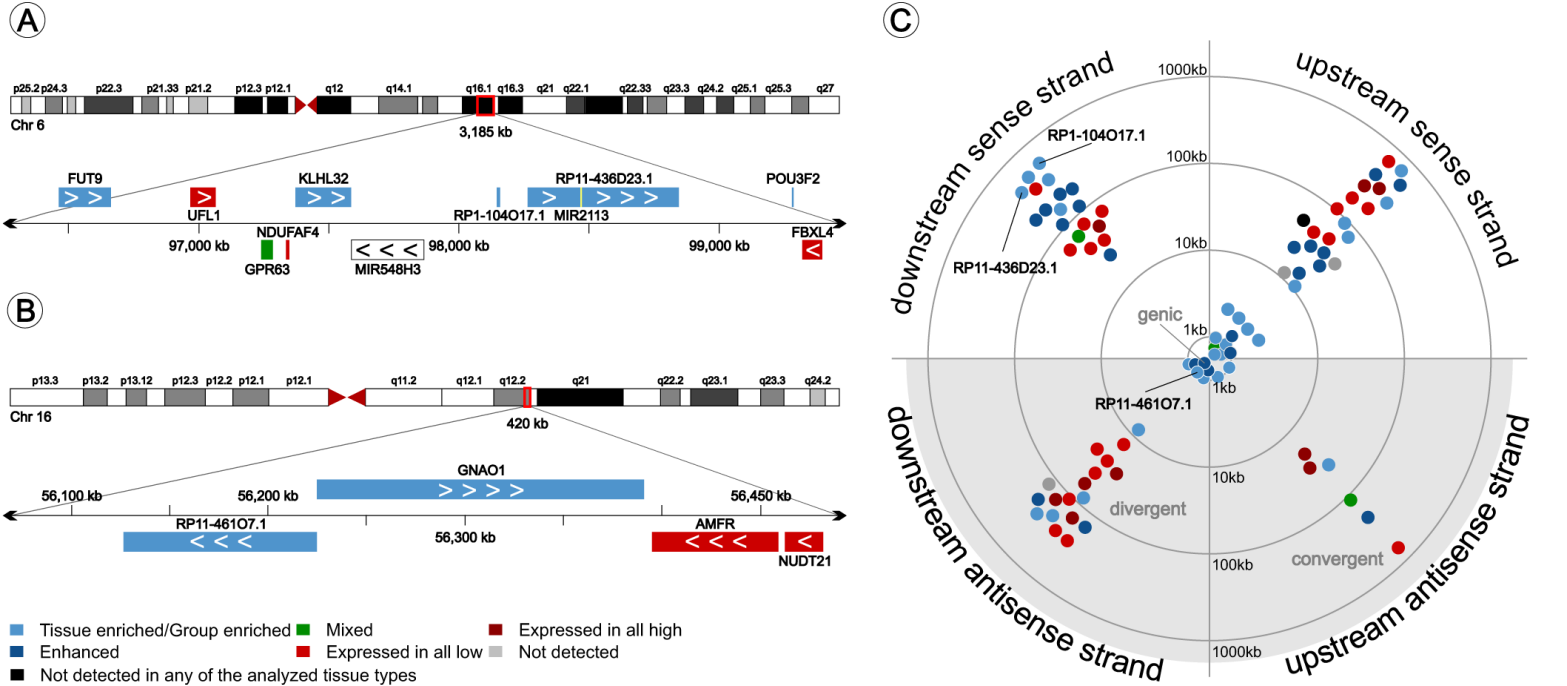
Genome mapping of brain-enriched lncRNA. All 87 brain-enriched lncRNAs were mapped to their genome location. Figure A gives an example of a cluster of brain-enriched genes located at position 6q16.1 containing the coding genes FUT9, KLHL32 and POU3F2 and the non-coding genes RP1-104017.1 and microRNA precursor RP11-436D23.1. Figure B shows an example of an lncRNA oriented divergent on the antisense strand from the protein-coding gene GNAO1. These genes possibly share their transcription initiation site. lncRNA gene orientation and color-coded expression characteristics of the nearest neighboring gene (distance < 1mb) are plotted in C. The majority of brain-enriched lncRNA are on the same strand as the nearest coding gene or divergent on the antisense strand. The majority (58%) of brain-enriched lncRNA are flanking brain-enhanced (dark blue) or brain-enriched (light blue) genes.

Based on Ensembl annotations, we analyzed the expression levels of 6,969 long non-coding RNAs (biotype lncRNA in Ensembl 73). We identified 2,959 lncRNA transcripts in the 27 tissue and organ types analyzed and found 661 lncRNA in frontal cortex. Out of these, we identified 49 brain-enriched and 38 group-enriched (including brain) lncRNAs (S2 Table). Detailed inspection of genomic regions containing brain-enriched lncRNAs and their closest neighboring protein coding genes revealed 1 intronic antisense and 2 exonic antisense genic brain-enriched lncRNAs and 83 intergenic in the sense (e.g. in Fig 8A) or antisense (e.g. in Fig 8B) direction of the nearest neighboring protein coding genes. One lncRNA (RP11-701H24.3) was found in a region containing several snoRNA genes and could not be linked to a protein-coding gene (S2 Table). We identified 6 brain-enriched microRNA-coding lncRNA including precursors for MIR4500, MIR2113, MIR137, MIR548N, MIR9-3 and MIR138-1 (S2 Table). Interestingly we found 51 (58%) of the lncRNA coding genes neighboring brain-enriched or-enhanced protein coding genes (Fig 8C), of which 20 (23%) lncRNA genes were found to flank transcription factor genes (NFIA, SOX1, SOX2, SOX8, SOX21, MEF2C, BHLHE22, MYT1L, ZIC1, ZIM2), including several homeobox genes (POU3F2, POU3F3, POU3F4, NKX2-2, NKX6-2, ZFHX4; S2 Table). These data illustrate the relationship between neighboring coding and non-coding genes and suggest a shared regulation of expression.

## Discussion

Due to its heterogeneity in function and cellular composition, multiple brain regions need to be analyzed to capture all transcripts expressed in the brain. Hawrylycz, Lein and colleagues were the first to carry out a comprehensive study on gene expression in 900 anatomically defined regions and sub-regions of the human brain [42]. Here we use a different approach and aim to identify brain-enriched transcripts and their cellular distribution. The presented results are based on the analysis of 95 samples from 27 tissue types and the detection and quantification of 18,363 (91%) protein-coding genes [9] and 2,959 (42%) non-coding genes expressed in the main organs of the human body. We identified coding and non-coding genes expressed in the brain with no or much lower expression in other organs (enriched/enhanced), and genes expressed in the brain with shared expression with other organs and tissue types (group-enriched). It seems relevant to continue this approach by zooming in on brain regions with cellular resolution combining cell selection and isolation techniques [43] with high throughput transcriptomics as has been executed in the mouse brain [25]. This will allow full characterization of cell types that populate the nervous system and identification of molecular networks based on cellular expression profiles. The integration of expression and distribution data will then advance the understanding of protein functions on a cellular and organ level.

First focus of this study was to generate a comprehensive list of genes with elevated expression in the human brain compared to analyzed peripheral tissues and to further characterize these brain-enriched protein-coding genes, by determining enriched biological functions and cellular expression. GO analysis of brain-enriched genes (including group-enriched), using all genes expressed in the brain as background, revealed many enriched GO-terms directly associated with development of the nervous system or synaptic signaling (Fig 2). The strong representation of developmental genes among brain-enriched genes is somewhat surprising, since no prenatal samples were included in our analysis. Detection of developmental genes can be explained by the sensitivity of the method, being able to detect traces of genes (e.g. transcription factors) highly expressed during development, then becoming low abundant after completion of cortocogenesis [44]. However, many developmental genes (e.g. adhesion molecules) have alternative functions in the adult brain. The enrichment of processes involved in chemical transmission is in line with the current understanding of brain physiology and is confirmed by IHC data. Of the 190 brain-enriched proteins examined, 65% was located in neuropil, containing synapses and glial endfeet (Fig 2). We then searched for currently uncharacterized brain-enriched genes expressed in various cortical cell types. We found that the majority of brain-enriched genes examined by immunohistochemistry is expressed in neurons, astrocytes or oligodendrocytes/Schwann cells. In contrast, genes expressed by microglia and endothelial cells, are expressed in multiple organ and tissue types. These finding are supported by cellular expression studies on the mouse cerebral cortex [25].

Do all proteins have a single molecular function and is adaptation of tissue and organs regulated by expression of genes and transcripts with specialized properties? The fact that we identified hundreds brain-enriched genes supports the hypothesis that organ function and properties are determined by the expression of proteins with specific molecular characteristics and cellular functions. However, the fact that we did not find many brain-specific genes, suggests a more context-dependent functional adaptation of proteins resulting in specific tissue and organ functions. This is supported by the many group-enriched genes expressed in brain and non-related tissue types (e.g testis and liver) or the expression of BBB proteins ABCB1 (P-gp) and ABCG2 (BCRP) in glandular cells in colon. We also explored NECAB1 splice variants expressed in brain and heart muscle. The fact that NECAB1 expressed in heart lacks the N-terminal calcium binding EF-hand motif suggests a different cellular function of this protein in heart muscle and brain.

An additional benefit of our approach is the possibility to identify and quantify the expression of lncRNAs. Here we analyzed the genomic organization and expression patterns of brain-enriched non-coding genes in relation to protein-coding neighboring genes. The results presented here suggest a consistent pattern of neighboring genes with similar enriched expression characteristics, and the existence of chromosomal ‘hot spots’ containing several brain-enriched coding and non-coding genes. For example a 3,185 kb region at position q16.1 of chromosome 6 (Fig 8A) contains several brain-enriched genes on the forward strand. Genome-wide association studies revealed a risk loci for bipolar disorder downstream of POU3F2 [45], and a region around KLHL32, GPR63 and NDUFA4 associated with Tourette syndrome and obsessive-compulsive disorder [46], indicating the importance of these enriched regions for establishing cortical functions. Our data also revealed a strong association of enriched lncRNA coding genes and transcription factor genes (S2 Table). These data support the hypothesis of locus control around transcription factor and homeobox genes by local chromatic modifications, which has been suggested to be a key process in switching from a transcription inactive to active state [47]. The current hypothesis is that a class of mainly intergenic lncRNAs are involved in the recruitment of chromatin modifiers to specific loci [48], which could explain lncRNA (HOTTIP) mediated regulate expression of neighboring HOXA genes [12], and could be a key process during organogenesis. It has recently become evident that transcription and epigenetic control involves chromatin modifications. Our results revealed an expression pattern that indicates regional regulation of clustered genes and a possible role of lncRNAs. However, which lncRNA and which molecular mechanisms are involved remains elusive.

In summary, we report for the first time the integration of deep sequencing transcriptomics of brain tissue homogenates with precise localization data on the single cell level using IHC results linking genes to proteins, cells and brain functions. This information will not only help to better understand brain evolution and physiology, but could also be utilized to direct the search for possibilities to intervene with disease processes.

## Methods

### Human tissue samples & Ethics

Human tissue samples used for protein and mRNA expression analyses were collected and handled in accordance with Swedish laws and regulation and obtained form the Department of Pathology, Uppsala University Hospital, Uppsala, Sweden as part of the sample collection governed by the Uppsala Biobank (http://www.uppsalabiobank.uu.se/en/). All human tissue samples used in the present study were anonymized in accordance with approval and advisory report from the Uppsala Ethical Review Board (Reference # 2002–577, 2005–338 and 2007–159 (protein) and # 2011–473 (RNA)), and consequently the need for informed consent was waived by the ethics committee. The use and analyses based these human tissues has previously been described [9].

### Transcript profiling (RNAseq)

All human tissues, including 3 frontal cortex samples (S1 Fig), were embedded in Optimal Cutting Temperature (OCT) compound and stored at—80°C. A hematoxylin-eosin (HE) stained frozen section (4μm) was prepared from each sample using a cryostat and the CryoJane Tape-Transfer System (Instrumedics, St. Louis, MO, USA). Each slide was examined by a pathologist to ensure proper tissue morphology and sample composition (white versus grey matter) and absence of evident pathological processes. Three sections (10μm thick) were cut from each frozen tissue block and collected into a tube for subsequent RNA extraction. The tissue was homogenized mechanically using a 3 mm steel grinding ball (VWR, Stockholm, Sweden). Total RNA was extracted from tissue samples using the RNeasy Mini Kit (Qiagen, Hilden, Germany) according to the manufacturer’s instructions. The extracted RNA samples were analyzed using either an Experion automated electrophoresis system (Bio-Rad Laboratories, Hercules, CA) with the standard-sensitivity RNA chip or an Agilent 2100 Bioanalyzer system (Agilent Biotechnologies, Palo Alto,CA) with the RNA 6000 Nano Labchip Kit. Only samples of high-quality RNA (RNA Integrity Number ≥7.5) were used in the following mRNA sample preparation for sequencing. mRNA sequencing was performed on Illumina HiSeq2000 and 2500 machines (Illumina, San Diego, CA) using the standard Illumina RNA-seq protocol with a read length of 2x100 bases. For sequencing, samples were multiplexed with at most 15 samples per lane, producing an minimum of 12 million and an average of 35.8 million mappable read pairs per sample (n = 95, S5 Table).

### Analysis of data

The raw reads obtained from the sequencing system were trimmed for low quality ends with the software sickle (https://github.com/najoshi/sickle), using a phred quality threshold of 20. All reads shorter than 54 bp after trimming were discarded. The processed reads were mapped to the GRCh37 version of the human genome with Tophat v2.0.3 [49]. Potential PCR duplicates were eliminated using the MarkDuplicates module of Picard 1.77 (http://picard.sourceforge.net/). To obtain quantification scores for all human genes, FPKM (fragments per kilobase of exon model per million mapped reads) values were calculated using Cufflinks v2.0.2 [50], which corrects for transcript length and the total number of mapped reads from the library to compensate for different read depths for different samples. The gene models from Ensembl build 73 [6] were used in Cufflinks. In addition to Cufflinks, HTSeq v0.5.1 was run to calculate read counts for each gene, which were used for analyses of differentially expressed genes using the DESeq package [51]. All data was analyzed using R Statistical Environment (http://www.R-project.org/) with the addition of package ‘gplots’ (http://CRAN.R-project.org/package=gplots). A network analysis (Fig 8) was performed using Cytoscape 3.0 [52]. For analyses performed in this study where a log2-scale of the data was used, pseudo-counts of +1 were added to the data set. The genetic landscape of brain-enriched lncRNA was examined using the integrative genomics viewer (IGV, Broad institute) and ensembl database.

### Specificity classification

There is currently no consensus on how to cluster and classify genes based on levels of expression in various organ and tissue samples. If a tissue-specific gene is only expressed in one type of tissue at any given time during life, it is necessary to define expression and to analyze all tissues at any developmental stage. Therefore, we believe the term ‘specific’ is misleading and prefer to use ‘enriched’, when comparing tissue types, and ‘enhanced’ when comparing mean global expression levels. The average FPKM value of all individual samples for each tissue was used to estimate the gene expression level. A cutoff value of 1 FPKM was used as the detection limit. Each of the 20,329 genes was classified into one of eight arbitrary categories based on the FPKM levels (Table 1).

**Table 1:** Classification of genes based on expression in 27 analyzed organ and tissue types.

Category	Description
**not detected**	FPKM <1
**expressed in all low**	detected in 27 tissues (FPKM > 1) and at least one tissue FPKM <10
**expressed in all high**	detected in 27 tissues and all tissues FPKM >10
**mixed**	detected in many (not all) tissues and not enhanced or enriched in any of the organ and tissue types
**group-enriched**	5-fold higher average FPKM level in a group of 2–7 tissues including frontal cortex compared to all other tissues
**enhanced**	5-fold higher FPKM level in frontal cortex compared to the average FPKM value of all 27 tissues
**enriched**	5-fold higher expression in frontal cortex compared to all other tissue and organ types
**highly enriched**	50-fold higher expression in frontal cortex compared to all other tissue and organ types

Criteria for the different RNA based gene categories based on the expression in 27 analyzed organs and tissue types.

### Gene ontology analysis

A gene ontology [53] analysis was performed using the GOrilla tool [54] in order to determine overrepresented GO categories in the gene set of 571 (557 associated to GO terms) tissue and group-enriched genes. A list of all 13,992 (12,872 associated to GO terms) genes expressed in the brain (FPKM >1) was used as the background list in GOrilla. Only enriched GO terms with a p-value <10–6 are included. For the cellular component analysis the GOSlim GOA associations were used to determine, whether genes were extracellular, intracellular or membrane bound. The number of genes for each term was counted, allowing a gene to be associated with more than one term.

### Tissue profiling

Tissue microarrays (TMA) containing triplicate 1-mm cores of 46 different types of normal tissue were generated as previously described [55]. TMA sections were immunostained as previously described [56]. Briefly, slides were deparaffinized in xylene, hydrated in graded alcohols and blocked for endogenous peroxidase in 0.3% hydrogen peroxide diluted in 95% ethanol. For antigen retrieval, a Decloaking chamber (Biocare Medical, Walnut Creek, CA) was used. Slides were immersed and boiled in Citrate buffer, pH6 (Lab Vision, Freemont, CA) for 4 minutes at 125°C and then allowed to cool to 90°C. Automated IHC was performed essentially as previously described [57], in brief, using an Autostainer 480 instrument (Lab Vision). Primary antibodies and a dextran polymer visualization system (UltraVision LP HRP polymer, Lab Vision) were incubated for 30 minutes each at room temperature and slides were developed for 10 minutes using Diaminobenzidine (Lab Vision) as chromogen. All incubations were followed by rinse in wash buffer (Lab Vision). Slides were counterstained in Mayers hematoxylin (Histolab, Göteborg, Sweden) and cover slipped using Pertex (Histolab) as mounting medium. Incubation with PBS instead of primary antibody served as negative control. The Aperio ScanScope XT Slide Scanner (Aperio Technologies, Vista, CA) system was used to capture digital whole slide images with a 20X objective. Whole slide images were de-arrayed to obtain individual images of each core. The outcome of immunohistochemical stainings in the screening phase, that included various normal tissues, was manually evaluated and scored by certified pathologists. In brief, the manual score of IHC-based protein expression was determined as the percentage of positive cells defined in different tissues: 0 = 0–1%, 1 = 2–25%, 2 = 26–75%, 3>75% and intensity of immunoreactivity: 0 = negative, 1 = weak, 2 = moderate and 3 = strong staining.

The immunohistochemical staining pattern was visually evaluated for antibodies targeting the genes of interest, and staining profiles were compared with RNA values in 27 analyzed tissues. Each antibody was scored for reliability, taking into account the effect of antibody titration on intensity of immunoreactivity. We considered an antibody to be reliable, if the staining pattern was (i) consistent with the expression profile, (ii) partly consistent with the RNA profile together with supporting western blot or (iii) was consistent when obtained with multiple antibodies recognizing different epitopes of the target protein. Only such antibodies were included in this study (S4 Table). In total 190 brain-enriched genes (127 brain-enriched and 63 group-enriched) as well as 130 genes expressed in all tissues with consistent immunohistochemistry data provided by at least two antibodies were selected. Neuronal-like and glia-like staining patterns and neuropil (including axons, dendrites, synapses and glial endfeet) were annotated manually. Staining patterns and annotations were verified using literature if available.

### Data availability

All the IHC and expression (FPKM values for all the samples) data are available for downloads without any restrictions (www.proteinatlas.org/about/download). The primary data (reads) are available through the Array Express Archive (www.ebi.ac.uk/arrayexpress/) under the accession number: E-MTAB-1733. The transcript profiling data (FPKM values) for each gene in each cell and tissue is available in the most recent version (v13) of the Human Protein Atlas (www.proteinatlas.org).

## Supporting Information

S1 Fig Overview of tissue selected for analysis of gene expression in the frontal cortex. Glass mounted brain sections from the frontal cortex (A) were analyzed to determine grey matter: white matter ratios (B-C). Grey matter areas are characterized by the presence of neurons with larger nuclei (D,F,H), whereas white matter structures do not contain neurons but do contain large numbers glial cells with smaller nuclei (E,G,I). Sample information is summarized in table. HE stainings of sample #1 could not be analyzed due to severe tissue damage during cryosectioning.
(EPS)

S2 Fig Cellular expression of human brain-enriched genes in mouse cortical cell populations. Cellular expression of human brain-enriched and group-enriched genes was extracted from the RNA-Seq transcriptome and splicing database of glia, neurons, and vascular cells of the cerebral cortex (http://web.stanford.edu/group/barres_lab/brain_rnaseq.html). The expression data was scaled and subjected to unsupervised hierarchical clustering using Euclidean distance to visualize clusters of genes more abundantly expressed in the different cell types. Similar to results for our IHC analysis we find the majority of brain-enriched genes to be expressed in a single cell-type. Note the overrepresentation of neurons, astrocytes and oligodendrocytes and underrepresentation of microglia and endothelial cells.
(EPS)

S1 Table Brain-enriched and enhanced genes (1113), contains FPKM values of frontal cortex and 26 peripheral organ and tissue types. (XLSX)

S2 Table Brain-enriched lncRNA. (XLS)

S3 Table Gene ontology analysis—GO terms and statistics. (PDF)

S4 Table Gene and Antibody information, summary of antibodies used for IHC analysis. (XLSX)

S5 Table Number of mapped reads for each sample analyzed (n = 95). (XLS)
