# Supplementary material for: Defining the Human Brain Proteome Using Transcriptomics and Antibody-Based Profiling with a Focus on the Cerebral Cortex
Source: PLoS One. 2015 Jun 15;10(6):e0130028. doi: 10.1371/journal.pone.0130028 (PMC4468152; doi:10.1371/journal.pone.0130028)
Supplement: S3 Table — (PDF) [file pone.0130028.s005.pdf]

	GO Term	Description	P-value	FDR q-value	Enrichment	B	b
Signaling	GO:2000311	regulation of AMPA selective glutamate receptor activity	1.98E-09	4.60E-07	**13.87**	15	9
	GO:0007214	gamma-aminobutyric acid signaling pathway	4.35E-09	8.97E-07	**13**	16	9
	GO:1900449	regulation of glutamate receptor signaling pathway	5.47E-08	9.33E-06	**10.4**	20	9
	GO:0060291	long-term synaptic potentiation	3.48E-07	5.44E-05	**10.27**	18	8
	GO:0048168	regulation of neuronal synaptic plasticity	1.67E-10	4.68E-08	**8.74**	37	14
	GO:0006836	neurotransmitter transport	9.39E-19	7.89E-16	**8.55**	73	27
	GO:0060079	regulation of excitatory postsynaptic membrane potential	8.78E-09	1.71E-06	**8.16**	34	12
	GO:0060078	regulation of postsynaptic membrane potential	2.22E-09	4.95E-07	**8.12**	37	13
	GO:0007269	neurotransmitter secretion	1.34E-11	4.43E-09	**7.86**	50	17
	GO:0007270	neuron-neuron synaptic transmission	1.85E-08	3.43E-06	**7.7**	36	12
	GO:0050807	regulation of synapse organization	1.69E-09	4.19E-07	**7.52**	43	14
	GO:0051588	regulation of neurotransmitter transport	4.14E-07	6.36E-05	**7.45**	31	10
	GO:0050806	positive regulation of synaptic transmission	2.38E-09	5.19E-07	**7.35**	44	14
	GO:0048167	regulation of synaptic plasticity	2.30E-16	1.67E-13	**7.09**	88	27
	GO:0051971	positive regulation of transmission of nerve impulse	4.58E-09	9.26E-07	**7.03**	46	14
	GO:0051899	membrane depolarization	9.37E-12	3.20E-09	**6.97**	63	19
	GO:0007268	synaptic transmission	1.40E-56	1.53E-52	**6.78**	341	100
	GO:0001505	regulation of neurotransmitter levels	9.67E-13	3.64E-10	**6.52**	78	22
	GO:0050804	regulation of synaptic transmission	3.66E-23	6.67E-20	**6.33**	157	43
	GO:0006814	sodium ion transport	2.27E-07	3.59E-05	**6.3**	44	12
	GO:0051969	regulation of transmission of nerve impulse	2.38E-21	2.89E-18	**5.74**	173	43
	GO:0006813	potassium ion transport	1.63E-09	4.25E-07	**5.62**	74	18
	GO:0023061	signal release	1.31E-10	3.77E-08	**5.45**	89	21
	GO:0050905	neuromuscular process	6.88E-07	1.02E-04	**5.27**	57	13
	GO:0007267	cell-cell signaling	1.00E-46	2.73E-43	**5.08**	482	106
	GO:0044700	single organism signaling	7.81E-47	4.26E-43	**4.99**	500	108
	GO:0023052	signaling	7.81E-47	2.84E-43	**4.99**	500	108
	GO:0042391	regulation of membrane potential	1.72E-13	7.22E-11	**4.68**	158	32
	GO:0015672	monovalent inorganic cation transport	3.10E-11	9.40E-09	**4.24**	158	29
	GO:0007154	cell communication	6.06E-40	1.32E-36	**4.15**	618	111
	GO:0030001	metal ion transport	1.41E-13	6.18E-11	**3.5**	297	45
	GO:0007186	G-protein coupled receptor signaling pathway	2.55E-13	9.93E-11	**3.28**	338	48
	GO:0006812	cation transport	1.13E-12	4.13E-10	**3.06**	377	50
	GO:0006811	ion transport	3.06E-17	2.38E-14	**2.8**	651	79
	GO:0043269	regulation of ion transport	7.95E-07	1.13E-04	**2.8**	231	28
	GO:0051046	regulation of secretion	7.56E-07	1.09E-04	**2.47**	328	35
	GO:0051049	regulation of transport	6.04E-07	9.16E-05	**1.85**	836	67
	GO:0044765	single-organism transport	1.67E-09	4.25E-07	**1.65**	1836	131
	GO:0006810	transport	1.78E-09	4.32E-07	**1.54**	2394	160
Neurological Process	GO:0007613	memory	1.94E-10	5.31E-08	**6.77**	58	17
	GO:0007612	learning	3.54E-14	1.61E-11	**6.76**	82	24
	GO:0031646	positive regulation of neurological system process	2.02E-08	3.68E-06	**6.34**	51	14
	GO:0031644	regulation of neurological system process	2.51E-20	2.49E-17	**5.43**	183	43
	GO:0007611	learning or memory	1.09E-14	5.69E-12	**5.31**	135	31
	GO:0050890	cognition	3.74E-15	2.27E-12	**5.15**	148	33
	GO:0030534	adult behavior	2.11E-08	3.78E-06	**4.84**	86	18
	GO:0007610	behavior	2.17E-22	3.39E-19	**4.31**	322	60
	GO:0007626	locomotory behavior	1.57E-08	2.96E-06	**4.26**	114	21
	GO:0050877	neurological system process	3.24E-19	2.95E-16	**3.59**	405	63
	GO:0007600	sensory perception	1.41E-08	2.69E-06	**3.14**	228	31
Development	GO:0007417	central nervous system development	2.22E-14	1.10E-11	**6.57**	88	25
	GO:0007409	axonogenesis	2.67E-09	5.71E-07	**5.17**	85	19
	GO:0048666	neuron development	1.73E-07	2.87E-05	**4.74**	78	16
	GO:0050770	regulation of axonogenesis	3.21E-08	5.66E-06	**4.48**	98	19
	GO:0048812	neuron projection morphogenesis	3.10E-09	6.52E-07	**4.11**	135	24
	GO:0007399	nervous system development	5.87E-15	3.38E-12	**4.04**	240	42
	GO:0010975	regulation of neuron projection development	1.76E-11	5.67E-09	**3.78**	208	34
	GO:0045664	regulation of neuron differentiation	1.01E-14	5.50E-12	**3.69**	288	46
	GO:0030182	neuron differentiation	1.24E-07	2.08E-05	**3.66**	139	22
	GO:0031344	regulation of cell projection organization	3.58E-11	1.06E-08	**3.45**	248	37
	GO:0048731	system development	1.40E-21	1.91E-18	**3.44**	504	75
	GO:0050767	regulation of neurogenesis	3.11E-14	1.48E-11	**3.36**	344	50
	GO:0051960	regulation of nervous system development	2.00E-15	1.36E-12	**3.34**	381	55
	GO:0010769	regulation of cell morphogenesis involved in differentiation	6.46E-07	9.66E-05	**3.13**	177	24
	GO:0048858	cell projection morphogenesis	7.16E-07	1.04E-04	**3.12**	178	24
	GO:0060284	regulation of cell development	2.64E-10	7.03E-08	**2.64**	437	50
	GO:0030030	cell projection organization	4.84E-08	8.38E-06	**2.39**	435	45
	GO:0048856	anatomical structure development	2.03E-15	1.30E-12	**1.9**	1764	145
	GO:0032502	developmental process	1.94E-09	4.60E-07	**1.48**	2834	182
Other	GO:0044708	single-organism behavior	2.04E-20	2.23E-17	**4.42**	277	53
	GO:0044057	regulation of system process	2.20E-13	8.91E-11	**3.24**	349	49
	GO:0003008	system process	2.40E-12	8.45E-10	**2.46**	648	69
	GO:0022610	biological adhesion	1.99E-07	3.25E-05	**2.09**	597	54
	GO:0007155	cell adhesion	1.99E-07	3.20E-05	**2.09**	597	54
	GO:0051234	establishment of localization	2.00E-09	4.55E-07	**1.54**	2437	162
	GO:0044763	single-organism cellular process	1.80E-11	5.61E-09	**1.26**	6675	365
	GO:0044699	single-organism process	5.62E-09	1.12E-06	**1.2**	7501	389
